# Pattern or process? Evaluating the peninsula effect as a determinant of species richness in coastal dune forests

**DOI:** 10.1371/journal.pone.0173694

**Published:** 2017-04-04

**Authors:** Pieter I. Olivier, Victor Rolo, Rudi J. van Aarde

**Affiliations:** Conservation Ecology Research Unit, Department of Zoology and Entomology, University of Pretoria, Pretoria, Hatfield, South Africa; Technion Israel Institute of Technology, ISRAEL

## Abstract

The peninsula effect predicts that the number of species should decline from the base of a peninsula to the tip. However, evidence for the peninsula effect is ambiguous, as different analytical methods, study taxa, and variations in local habitat or regional climatic conditions influence conclusions on its presence. We address this uncertainty by using two analytical methods to investigate the peninsula effect in three taxa that occupy different trophic levels: trees, millipedes, and birds. We surveyed 81 tree quadrants, 102 millipede transects, and 152 bird points within 150 km of coastal dune forest that resemble a habitat peninsula along the northeast coast of South Africa. We then used spatial (trend surface analyses) and non-spatial regressions (generalized linear mixed models) to test for the presence of the peninsula effect in each of the three taxa. We also used linear mixed models to test if climate (temperature and precipitation) and/or local habitat conditions (water availability associated with topography and landscape structural variables) could explain gradients in species richness. Non-spatial models suggest that the peninsula effect was present in all three taxa. However, spatial models indicated that only bird species richness declined from the peninsula base to the peninsula tip. Millipede species richness increased near the centre of the peninsula, while tree species richness increased near the tip. Local habitat conditions explained species richness patterns of birds and trees, but not of millipedes, regardless of model type. Our study highlights the idiosyncrasies associated with the peninsula effect—conclusions on the presence of the peninsula effect depend on the analytical methods used and the taxon studied. The peninsula effect might therefore be better suited to describe a species richness pattern where the number of species decline from a broader habitat base to a narrow tip, rather than a process that drives species richness.

## Introduction

The peninsula effect predicts a decrease in the number of species from a broad peninsula base towards a narrow distal tip [1], and is one of the classic biogeographic forces that have been proposed to explain gradients in species richness [2]. However, evidence supporting the peninsula effect has been mixed [3], and several studies have concluded alternative drivers of species richness on peninsulas [4, 5].

Since Simpson [1] first proposed the peninsula effect, it has been investigated in more than 80 studies (see [6] for a full review), which encompassed a range of geographic regions [7, 8, 9, 10], taxa, [11, 12] and peninsula types (continental and habitat peninsulas; [11, 13]. However, when reviewing a subset of these, Jenkins & Rinne [14] found that the peninsula effect only explained species richness trends in about half the cases (18 out of 37). For instance, on continental peninsulas, there was strong evidence for a peninsula effect in Iberian butterflies [15], but not for scorpions on the Baja California peninsula [4]. Similar idiosyncratic results have also been found in habitat peninsulas. For example, Tackaberry and Kellman [16] reported no peninsula effect in tropical forest trees, while Tubelis *et al*. [12] found a marked peninsula effect in eucalypt forest birds. Moreover, various other species richness patterns have been recorded for habitat and continental peninsulas. These include high species richness in the centre of peninsulas, compared to the base and tip [10], reverse gradients with species richness increasing from the peninsula base to the tip [17], and peninsulas with no distinct species richness patterns [14].

The idiosyncrasies that mark studies of the peninsula effect may be related to at least four confounding factors. First, a number of different methods have been used to assess the presence of a peninsula effect. These relate to units of measurements (e.g. species richness versus species density), but also to analytical approaches. For instance, most studies on the analysis of geographical gradients use regression models to determine trends in species richness (e.g. [18]. However, compared to spatial models (i.e. those that takes into account the spatial structure) (e.g. [19]), non-spatial regression models can generalize species richness trends; thereby influencing conclusions on the presence of the peninsula effect [20]. Second, local variations in habitat conditions along peninsulas may drive gradients in species richness [5, 3, 14, 21]. These may include, amongst others, differences in soil types, topography, energy, and disturbance regimes [22, 23]. Third, because studies on the peninsula effect mostly investigate species richness patterns across regional scales, trends in species richness might be incorrectly attributed to a peninsula effect, when it is really caused by climate [24]. Lastly, the peninsula effect likely affect taxa with different life history strategies differently [3, 11]. For instance, volant taxa, such as birds, are more likely to exhibit a peninsula effect than non-volant taxa, such as plants [3, 14]. Studies that fail to take into account these confounding factors may incorrectly conclude the presence or absence of a peninsula effect.

In this study we investigated the peninsula effect as a determinant of tree, millipede and bird species richness in sub-tropical coastal dune forests in South Africa. We overcome the constraints listed above by using two different analytical approaches (spatial and non-spatial regression) to investigate the peninsula effect in three taxa with different life history strategies. These taxa represented three trophic levels: primary producers (trees), consumers (birds) and decomposers (millipedes). We then test if climate and/or local habitat conditions could explain the presence of the peninsula effect in each of the three taxa. We aim to answer three questions: 1) Do conclusions on the presence of the peninsula effect depend on the analytical method used? 2) Are some taxa more likely to display a peninsula effect than others? And 3) Could the peninsula effect be explained by differences in climate or local habitat conditions? We expect that conclusions on whether or not a peninsula effect is present will depend on the analytical method used. We therefore are more likely to conclude the presence of the peninsula effect from non-spatial than spatial regression models. We furthermore expect that if a peninsula effect was present, birds would exhibit the strongest peninsula effect, as they were the most volant taxa in this study, whereas trees would exhibit the weakest peninsula effect [14]. Finally, we expect that variation in climate will explain the peninsula effect, given the presence of the tropical-temperate climatic gradient along the South African east coast [25].

## Materials and methods

Permits for field surveys were granted by Ezemvelo KZN Wildlife and iSimangaliso Wetland Park. Tongaat Hullet Estate provided permission that allowed surveys within forests located on their estates. Sampling procedures were reviewed and approved as part of obtaining the field permits.

### Study area

Coastal dune forests form part of the Indian Ocean Coastal Belt Forests [25] and are the southernmost, hence marginal, outlier of East African Tropical Coastal Forest, which extends along the southern Somalian, Kenyan, Tanzanian, Mozambican and South African coasts [26].

These forests established on porous and leached sand deposits left by a regressing Indian Ocean during the end of the last glacial period 8000 to 10000 years ago [27]. Changes in climate likely led to the expansion and contraction of dune forests until around 6000 BP [27; 28]. Thereafter, warmer and wetter conditions led to a period of forest radiation, which allowed the colonization of South African dune forests by Afrotropical forest faunas [29]. Given the relatively recent establishment of coastal dune forests, it has not yet experienced a climatic extinction filtering event [29]. However, people have exploited these forests since the early Iron Age (around 800 BP). Their impact intensified with the rise of the Zulu kingdom (early 1800s) and the arrival of Europeans (mid 1800s (see [30] and references therein). Historical records do not provide detailed information on how much of these forests have been cleared during the last two centuries, but models suggest that 82% of coastal forests may have been lost [30]. At present, dune forests form part of a centre of plant endemism [31], a biodiversity hotspot [32] as well as two critically endangered eco-regions [33]. They also harbour a number of species of conservation concern [34] as well as an extinction debt incurred from past forest losses [30].

In South Africa, coastal dune forests resemble a habitat peninsula that stretches southwards from the northern tropics towards the more temperate south. The habitat peninsula differ from most other habitat peninsulas that have been studied in the literature, mainly because it does not ‘penetrate’ into another ecosystem that functions as a wraparound ‘ocean’ on all sides. The studied habitat peninsula was adjoined by human land use types in the west and the Indian Ocean in the east. We assume that this difference would not affect our conclusions on the peninsula effect for two reasons. First, the Indian Ocean adjoined the habitat peninsula along the entire eastern boundary. The influence of the ocean on the forest peninsula would therefore be similar along the 150km extent. Second, the different matrix habitats that adjoin the peninsula on the western boundary are unlikely to influence forest assemblages differently because species from adjacent matrix habitats seldom spillover into forests, irrespective of whether the matrix is transformed (human land-use types) or natural (grasslands or woodlands) [35]. Rather, forest species spillover into the adjacent matrix habitats, which may boost species diversity within these habitats [35]. If edge effects were present, it was therefore likely to be similar for the entire peninsula. However, because the width of the peninsula decreased towards the south, and assuming equal edge effects, the forest core in the south would be smaller than the forest core in the north. The difference in the availability of forest core habitats may therefore influence the diversity of forest species. In summary, both the Indian Ocean and the transformed matrix have similar functions—to hinder the spillover of species into the forest. Thus, despite being two distinctive units, we assume that these different contact zones would not cause variation in the forest assemblages we studied.

**Fig 1:**
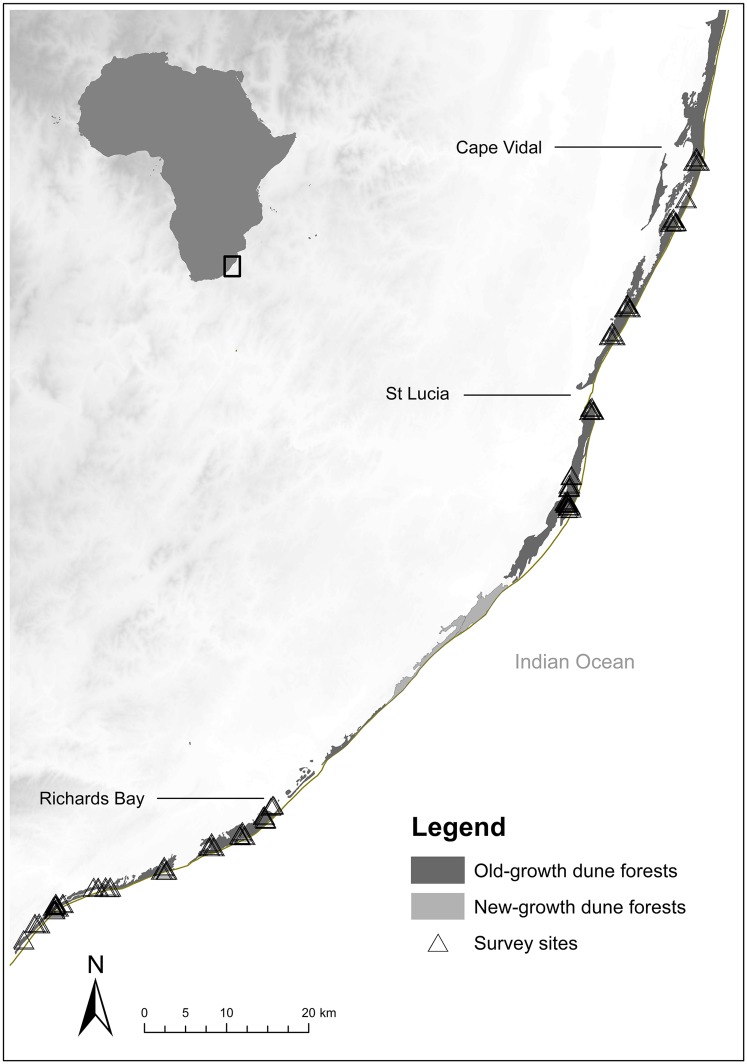
The distribution of the coastal dune forest (habitat peninsula) along the east coast of South Africa. Dark grey areas represent old-growth forests, in which this study was conducted, and light grey areas represent regenerating new-growth forests. All survey sites are indicated by triangles.

The forest, termed here as the ‘peninsula’, was wider in the north (maximum width of 1.25 km) than in the south (maximum width of 150 m). The survey area stretched from the peninsula base (Cape Vidal, 28°13´S, 32°55´E) in the north to the peninsula tip (Umlalazi Nature Reserve, 29°24´S, 31°48´E), some 150 km to the south (Fig 1).

### Field surveys

#### Tree surveys

We surveyed 81 tree plots from September 2012 to November 2012. Tree plots were 16 x 16 m and were located randomly along the forest peninsula. Within each tree plot, we recorded and identified all individuals taller than 30 cm.

#### Millipede surveys

Millipede sampling took place in 102 randomly located plots during January and February from 2002 to 2012. Surveys were conducted between 0500 h and 1000 h as millipedes are most active during this time of the day [36]. Transects were 6 x 16 m and were systematically searched for millipedes by three trained observers. Observers recorded all adult millipede species from the forest floor to those found in trees up to a height of 1.7 m. The soil layer was not included in the search as it mostly contains juveniles that are difficult to identify [37].

#### Bird surveys

Birds were sampled using point counts at 152 randomly selected points. Three trained and experienced observers surveyed points between 0400 h and 0900 h from November 2011 to March 2012. At each point, birds were allowed two minutes to resettle after the arrival of the observer. Following this, all birds seen or heard were recorded for a period of 10 minutes. For each encounter, estimated distances from the observer to the bird were recorded with a digital rangefinder (Nikon Laser 550As; Nikon, Tokyo, Japan). Birds seen flying over the canopy were excluded. No surveys were conducted in rainy or windy weather conditions.

### Environmental variables

At each sampling point, we sourced environmental variables that could potentially influence patterns of species richness (Table 1). Climatic variables (average annual temperature and rainfall) were obtained from the global database WorldClim [38]. This database provides global coverage of climatic data at one km^2^ resolution. Climatic variables, such as precipitation and temperature, have been shown to have direct effects on the species richness of trees and birds [24, 39], and may affect the distribution of millipedes [40]. To account for local factors that can potentially modulate overall climatic variables, we computed a topographic wetness index (TWI) based on the digital elevation model at 30 m resolution from [41]. This index quantifies the capacity that an area has to accumulate water. Low values are typical of steep areas and indicate low water accumulation capacity, whereas high values are typical from flat areas prone to accumulate water. TWI was calculated for each pixel as:

$$TWI=ln\left(\frac{As}{\beta }\right)$$

where *As* is the drainage area (m^2^) and β is the slope in radians [42]. TWI has been shown to be a good predictor of species occurrence of birds [43] and trees [44] and could be especially important in dune forests given the variable topography underlying the distribution of these forests [25]. Finally, we calculated landscape structural variables to assess the effect of landscape quality and configuration at each sampling point. To generate homogeneous spatial units across the study area, we created a grid of hexagonal units of 10 ha each. Based on a forest cover layer [45], we computed the area of forest and an area weighted mean shape index (AWMSI) for each hexagon. This index relates to shape complexity and is computed as the sum of each patch's perimeter, divided by the square root of the patch area. Circular patches have an AWMSI value close to one and it increases with patch shape irregularity. Total area and AWMSI has been shown to be a good predictor of bird and plant species richness patterns [46]. Landscape structural variables were computed using Patch Analyst [47]. All variables were computed using ArcGIS 10.3.1 [48].

**Table 1:** Description of the predictor variables used in spatial and non-spatial models to determine the effects of climate, local habitat conditions and the peninsula effect on tree, millipede and bird species richness in a coastal dune forest habitat peninsula.

Environmental variable	Description
Forest Area (ha)	Amount of forest within each hexagon
	(Range = 2.6–10).
Area Weighted Mean Shape Index (AWMSI)	Shape complexity of forest patches within each hexagon
	(Range = 1–2.6)
Precipitation (mm)	Mean annual precipitation
	(Range = 1118–1328)
Temperature (°C)	Mean annual temperature
	(Range = 21.14–21.84)
Terrain Wetness Index	Capacity of an area to accumulate water
	(Range = 0–6.6)

### Data analyses

We used two approaches to assess if a peninsula effect is present in coastal dune forests. First, we used generalized linear mixed models (GLMMs) to determine if the number of species decreased from the base to the tip of the dune forest peninsula. We did so for each taxon, by including species richness as the response variable and position (i.e. distance from the base in km) as the predictor variable. For birds, we only included forest-dependent species in our analyses. We defined forest-dependent species as those that live and reproduce only in forest habitats based on [49]. For trees and millipedes, all species were included in our analyses because species recorded in the forest did not occur in the adjacent matrix habitats. We assumed that the response variable (count data) was Poisson distributed (log-link) and included transect as a random effect. We only included an observation-level random effect in the model for tree species richness as the models for birds and millipedes did not show overdispersion. We call these models non-spatial because they do not include the coordinates of each sampling point. Second, we used a trend surface analysis to determine if species richness patterns were spatially structured [19]. Trend surface analysis assesses the presence of spatial patterns by using a regression model calibrated over the entire study area. The procedure aims to predict a response variable (i.e. species richness) based solely on the geographic coordinates X and Y of the sampling sites. If a peninsula effect is present in coastal dune forests, species richness patterns should show a spatial structure. We call these models spatial because they include the coordinates of each sampling point. To facilitate the comparison of species richness levels among taxa, we calculate z-values of species richness per taxa and used them as response variables in the trend surface analysis. Positive z-values indicate high number of species, while negative z-values indicate low number of species.

To assess if environmental variables could explain species richness patterns obtained from spatial and non-spatial models, we used linear mixed models that included transect as a random variable. Using regression models to assess the influence of environmental variables on spatially structured data may result in an overestimation of the importance of the environmental variables [20]. To limit this bias, we detrended species richness by computing the residuals from the trend surface equations and used the residuals as response variables in the linear mixed models. We used the same approach for the non-spatial models. To evaluate which environmental variables contributed consistently across taxa we followed a model averaging approach. For both spatial and non-spatial models, regression coefficients of each explanatory variable were averaged across all competing models where ΔAICc < 2. For each competing model, Akaike Information Criterion weight (AICc-wi), which represents the likelihood of a given model relative to all other models, was generated. An importance value of each explanatory variable was calculated by adding the AICc-wi values of the competing models in which the predictor was present. Importance values vary between zero (low importance) and one (high importance). Environmental variables showed a variance inflation factor below two in all competing models, indicating limited collinearity among them. All analyses were carried out in the R statistical environment [50] using the packages lme4 [51] and MuMln [52].

## Results

During our surveys, we recorded 15 978, 2518 and 1539 trees, millipedes and birds respectively. These comprised 160 tree species, 25 millipede species, and 59 bird species.

**Fig 2:**
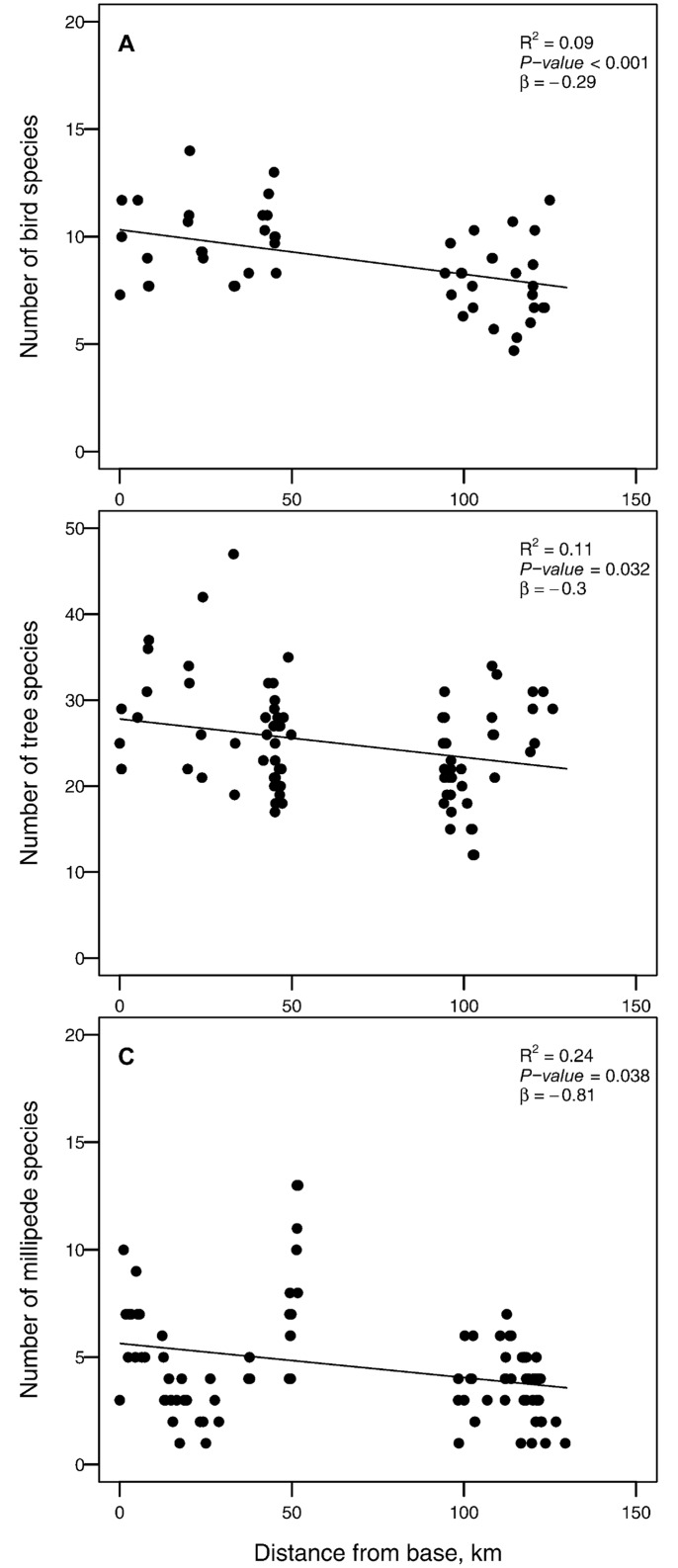
Non-spatial regression that illustrate the relationship between species richness of (A) tree, (B) millipede and (C) bird species and distance from the base of the coastal dune forest peninsula to the tip. R^2^ values represents the variability explained by position without including the random effects (i.e. marginal R^2^).

GLMMs (non-spatial models) suggest that species richness for all three taxa declined significantly with increasing distance from the base to the tip of the peninsula, as predicted by the peninsula effect (Fig 2). This decreasing trend was most pronounced for millipedes with a predicted 66% reduction in species richness per 100 km, while trees (29%) and birds (26%) showed weaker, but similar responses.

**Fig 3:**
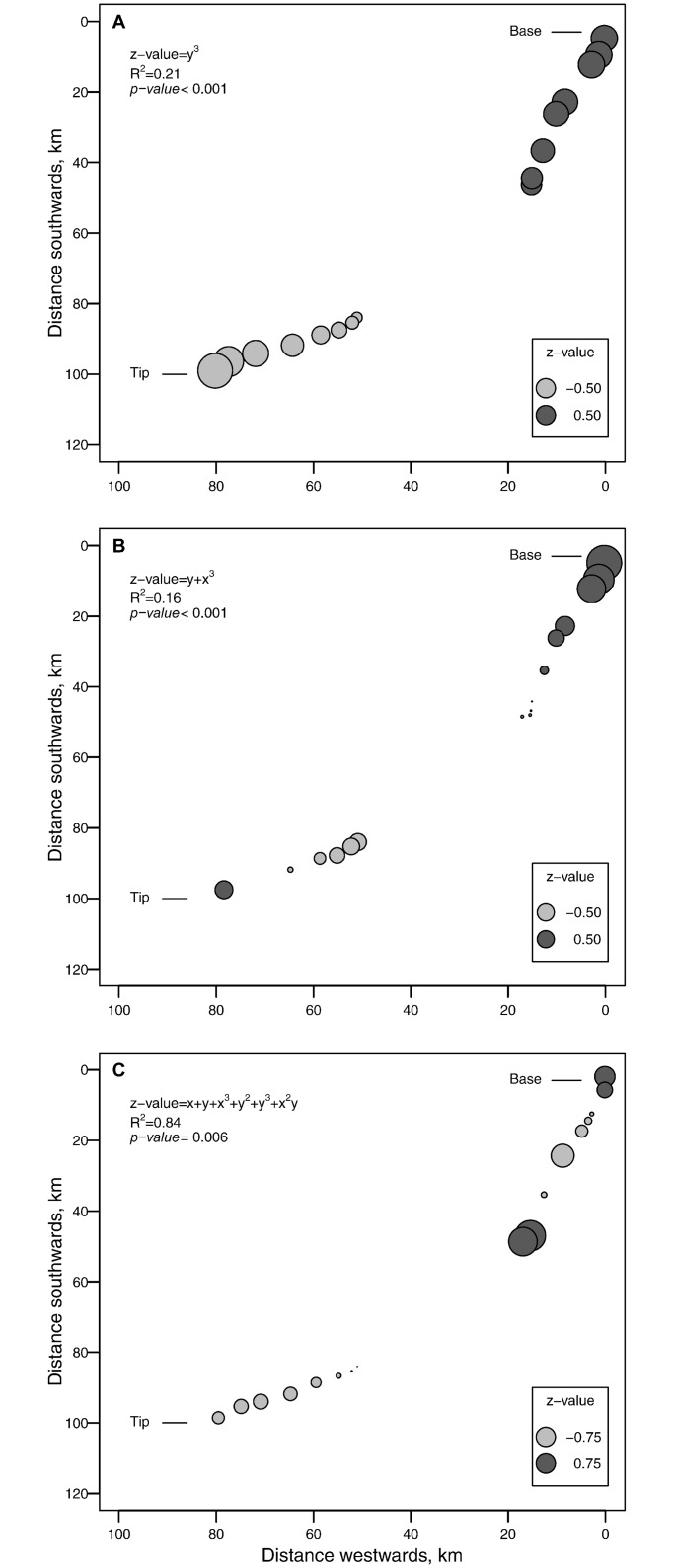
Spatial regression (trend surface models) that illustrate predicted z-values of species richness of (A) bird, (B) tree and (C) millipede of each transect sampled along the coastal dune forest peninsula.

However, trend surface analyses of z-values of species richness (spatial models) did not produce a similar pattern (Fig 3). Although we found significant trend surface models for all three taxa, only birds showed a consistent reduction in species richness from the peninsula base to the tip. For millipedes and trees, however, z-values increased in the centre of the peninsula for millipedes but at the tip of the peninsula for trees. The trend surface model of millipedes explained the highest amount of variability among taxa (R^2^ = 0.84), followed by birds (R^2^ = 0.22) and trees (R^2^ = 0.61).

For both spatial and non-spatial models, environmental factors significantly explained the remaining variation of species richness after they were detrended (Table 2). Original values of species richness and detrended residuals were significantly and positively related for spatial (Pearson correlation: r = 0.9 *P-value* < 0.001; r = 0.87 *P-value* < 0.001; r = 0.71 *P-value* < 0.001 for tree, bird and millipede respectively) and non-spatial models (Pearson correlation: r = 0.88 *P-value* < 0.001; r = 0.88 *P-value* < 0.001; r = 0.82 *P-value* < 0.001 for tree, bird and millipede respectively). This indicates that similar biological interpretations can be made from detrended z-values and the original values of species richness.

**Table 2:** Coefficient estimates and adjusted Standard Errors (SE) of environmental variables (forest area, AWMSI, temperature, rainfall and TWI) after model averaging of GLMMs to explore their relationship with tree, millipede and bird species richness. The response variables in all models were detrended species richness (i.e. residuals from spatial and non-spatial models). Estimates represent standardize values and, therefore, they are in a comparable scale. The relative importance of predictor variables was calculated as the sum of AICc-wi values of the competing models in which the predictor was present. Significant predictors at P < 0.05 are depicted in bold and marginally significant (0.05 < P < 0.1) in italics.

		Estimate	SE	*P-value*	Importance	Num. Models*
Spatial						
Tree	AWMSI	0.254	0.100	**0.011**	1.00	3 (3)
	TWI	-0.203	0.094	**0.031**	1.00	3 (3)
	Forest Area	0.066	0.098	0.497	0.22	1 (3)
	Temperature	-0.065	0.102	0.522	0.21	1 (3)
Bird	Forest Area	-0.232	0.120	*0*.*054*	1.00	4 (4)
	TWI	0.252	0.121	**0.038**	0.77	3 (4)
	Temperature	-0.108	0.118	0.358	0.18	1 (4)
	AWMSI	-0.108	0.120	0.369	0.17	1 (4)
Millipede	Rainfall	0.092	0.134	0.495	0.17	1 (4)
	Temperature	-0.050	0.092	0.585	0.15	1 (4)
	TWI	0.031	0.061	0.611	0.15	1 (4)
	Forest Area	-0.034	0.073	0.645	0.15	1 (4)
Non-Spatial						
Tree	TWI	-0.202	0.095	**0.033**	0.89	4 (4)
	AWMSI	0.134	0.097	0.164	0.51	3 (4)
	Temperature	-0.106	0.095	0.264	0.34	2 (4)
Bird	Forest Area	-0.169	0.083	**0.043**	1.00	3 (3)
	TWI	0.181	0.084	**0.031**	0.76	2 (3)
	AWMSI	-0.081	0.083	0.328	0.25	1 (3)
Millipede	Rainfall	-0.160	0.099	0.107	0.81	3 (4)
	Temperature	0.140	0.171	0.412	0.21	1 (4)
	TWI	0.043	0.073	0.551	0.17	1 (4)

*Number of times in which a predictor appears in the set of competing model (AICc < 2) per taxa.

Numbers in brackets indicates the number of competing models.

Furthermore, climate and local environmental factors had the same effect on detrended values of spatial and non-spatial models. Among environmental factors, landscape structural variables and terrain wetness index (TWI) showed the highest effects on detrended values (Table 2). However, the sign of the relationship depended on the taxa considered. We observed a negative relationship between TWI and detrended values for trees but positive for birds. These patterns suggest that valleys of zones with higher accumulation of water (i.e. high TWI values) had a low number of tree species, but a high number of bird species. Detrended values of bird species richness were negatively related to the forest area. Whereas shape complexity of forest patches was positively related to detrended values of tree species richness. None of the environmental factors considered was a significant predictor of detrended values of millipede species richness. Climatic variables did not show any significant relationship among taxa. Both temperature and rainfall were significantly correlated with peninsula position (r = 0.64 *P-value* < 0.001; r = - 0.30 *P-value* < 0.001, respectively).

## Discussion

The general applicability of the peninsula effect as a biogeographic phenomenon has always been uncertain [14, 53]. Our analyses highlight this uncertainty—conclusions on the presence of the peninsula effect depended on the analytical methods used and the taxon studied. Moreover, in coastal dune forests, variations in local habitat conditions mostly affected species richness patterns. It might therefore be more useful to refer to the ‘peninsula pattern’ rather than the ‘peninsula effect’ as our results suggests that the peninsula is not a process that drives species richness, but rather describes a species richness pattern where the number of species decline from a broader base to a narrow tip.

We found support for our first expectation that conclusions on the presence of the peninsula effect depend on the analytical method used. Had we only used non-spatial regression to investigate the peninsula effect, we would have concluded that such an effect was present for all three taxa, with millipedes showing the strongest effect. However, because this approach only use distance from the peninsula base (i.e. peninsula position) as a predictor, it may mask fine scale patterns in species richness. This becomes evident when we examine the spatial structure of species richness patterns. Based on our trend surface models, only birds showed a consistent decrease in the number of species from the peninsula base to the peninsula tip. Millipede species richness increased near the centre of the peninsula, while tree species richness increased near the tip. Invoking the peninsula effect as an explanation of species richness patterns for these two taxa therefore does not hold, even though linear models suggest it does. Linear regression models may therefore not be the correct method to investigate the peninsula effect. Because it does not account for the spatial structure among sampling points, it may distort trends in species richness. Trend surface models, on the other hand, use the geographic coordinates of sampling sites to model species richness [19]. This method is sensitive to increases or decreases in species richness at different locations across a spatial gradient, and may therefore present a more sensible method for future studies to investigate the peninsula effect. Notably, however, we found similar effects of climate and local environmental factors on detrended values of spatial and non-spatial models. In other words, based on our models, the factors that gave rise to the observed species richness patterns were the same, regardless of model type. This likely happened because peninsula position was correlated with regional factors such as climate. Because regional (i.e. climatic) variation has been accounted for in both models, only the effects of local environmental variables remain. This highlights the potential confounding effect between broad-scale climatic variation and the peninsula effect.

Species richness patterns for the three taxa considered in our study varied across the dune forest peninsula. Only birds exhibited a peninsula effect, which support our expectation that more volant taxa would be more likely to exhibit a peninsula effect than less volant ones. Volant taxa have few constraints on their dispersal and may best illustrate recolonization patterns after disturbance events or climatic perturbations [54]. For instance, in coastal dune forests, birds may exhibit a peninsula effect because of the historic dispersal patterns of Southern African faunal communities [28, 29]. Dune forests in South Africa were colonised by Afrotropical forest faunas that dispersed along a coastal route southwards after the last glacial maximum (*c*. 18000 yr BP) [29]. Because dispersing tropical species may still be colonizing this forest peninsula, species richness decreases from the northern tropical base to the southern tip. However, our results also suggest that local habitat conditions may cause the observed peninsula effect. Bird species richness was strongly associated with the topographic wetness index—species richness was high when there was a high accumulation of water, but lower when water accumulation was low. In dune forests, water accumulation is generally higher within dune valleys than along dune crests. Valleys are also more sheltered from strong winds, as well as salt spray from the ocean and may provide a more stable environment that harbours a high diversity of birds. The observed peninsula effect may therefore have little to do with a regional biogeographic process, but rather with the ability of birds to shift their distributions to track changes in habitat conditions.

Although the pattern for trees were similar to that recorded for birds, tree species richness increased at the tip of the peninsula. Many tropical tree species reaches their southernmost distributional limit in coastal dune forests, while a number of temperate species reach their northernmost distributional limit [34]. The presence of a few temperate species may therefore lead to the observed increase in tree species richness at the peninsula tip. However, if this were the case, climatic variables (e.g. temperature and precipitation) should play an important role in determining tree species richness. Yet, contrary to our expectation, climate did not significantly affect species richness in any of the three taxa. For trees, the spatial configuration of forest habitat (e.g. area weighted mean shape index) and local environmental conditions at survey sites (topographic wetness index) significantly affected species richness. There could be two explanations for this finding. First, because temperature and rainfall were significantly related to position along the peninsula, the trend surface analysis may have accounted for the variation in climatic conditions along the peninsula. Climatic variations operate at coarse spatial scales, and once this has been accounted for, the influence of local environmental conditions becomes clearer. Second, different human land-use types (i.e. urban and rural settlements, sugarcane and Eucalyptus plantations) adjoin the coastal dune forest peninsula. Disturbances associated with such human land-use types (e.g. edge effects) may alter tree species richness at local scales [55]. For instance, edge effects associated with high contrast land-use types may lead to increase in invasive plant species, disturbance favouring early successional trees and climbers, coupled with a correlated decline of old-growth trees [56]. If this is the case, the role of local disturbances in determining species richness, may prevail over regional processes like climate and the peninsula effect [57].

None of the variables included in our analysis had any significant effect on millipedes. This is perhaps not surprising as millipedes are sensitive to desiccation in dry conditions due to the permeability of their cuticle layer and therefore select for areas with high humidity and moderate temperatures near the forest floor [58]. Variables that relate to coarse scale climatic conditions or the spatial configuration of forest habitats may therefore have little explanatory power. Millipedes may also display behavioural responses to fine-scale temperature and moisture conditions [59]. For instance, millipede activity generally decreases during dry conditions and under high temperatures, which may influence their detectability during surveys and our interpretation of recorded species richness patterns [60].

The high species diversity of coastal dune forests is under pressure from commercial agriculture, urban expansion, and mining activities [34]. As a result, the once continuous forest peninsula are becoming more fragmented than what was the case in the past, which could influence the occurrence of forest species [61]. Such a decrease in connectivity may mean that a habitat archipelago model may eventually be used to describe region dynamics here. Nevertheless, conservation efforts that focuses on these forests might benefit from our conclusions, as the number of species present along the current dune forest peninsula could influence the location of protected areas. For example, for birds, protected areas at the base of the forest peninsula will cater for more species than those at the peninsula tip; however, this will not be the case for trees or millipedes. Protected areas at the base of the dune forest peninsula might aim to encompass large areas of regular shape, which maximises the core area, while conservation strategies at the tip might focus directly on the prevention of species level extinctions [6]. Moreover, the need for restoration efforts in coastal dune forests has become more relevant, specifically because of dune mining along the South African east coast [62]. Although coastal dune forest taxa do not necessarily display a peninsula effect, restoration efforts should consider gradients in species richness when evaluating the development of new-growth forests. Because species richness differs along the dune forest peninsula, it may not make sense to use forest sites located at the base of the peninsula as benchmarks to evaluate regenerating sites at the tip.

## Supporting information

S1 Dataset This file contains the data from the article “Pattern or process? Evaluating the peninsula effect as a determinant of species richness in coastal dune forests” by Pieter I. Olivier, Victor Rolo, and Rudi J. van Aarde. (CSV)
