# Correction: Pattern or process? Evaluating the peninsula effect as a determinant of species richness in coastal dune forests

**DOI:** 10.1371/journal.pone.0197623

**Published:** 2018-05-14

**Authors:** Pieter I. Olivier, Victor Rolo, Natasha Visser, Rudi J. van Aarde

Natasha Visser is not included in the author byline. She should be listed as the third author and her affiliation is 1: Conservation Ecology Research Unit, Department of Zoology and Entomology, University of Pretoria, Pretoria, Hatfield, South Africa. The contributions of this author are as follows: Data curation, Investigation, Methodology, Writing–Original Draft Preparation.

The correct citation is: Olivier PI, Rolo V, Visser N, van Aarde RJ (2017) Pattern or process? Evaluating the peninsula effect as a determinant of species richness in coastal dune forests. PLoS ONE 12(4): e0173694. https://doi.org/10.1371/journal.pone.0173694
